# Phenotype and Function of CD209^+^ Bovine Blood Dendritic Cells, Monocyte-Derived-Dendritic Cells and Monocyte-Derived Macrophages

**DOI:** 10.1371/journal.pone.0165247

**Published:** 2016-10-20

**Authors:** Kun Taek Park, Mahmoud M. ElNaggar, Gaber S. Abdellrazeq, John P. Bannantine, Victoria Mack, Lindsay M. Fry, William C. Davis

**Affiliations:** 1 Department of Veterinary Microbiology and Pathology, Washington State University, Pullman, WA 99164, United States of America; 2 Department of Veterinary Microbiology, College of Veterinary Medicine, Seoul National University, Seoul 151–742, Republic of Korea; 3 Department of Microbiology, Faculty of Veterinary Medicine, Alexandria University, Egypt; 4 USDA, ARS, National Animal Disease Center, Ames, Iowa, United States of America; 5 USDA, ARS, Animal Disease Research Unit, Pullman, WA 99164, United States of America; Institut National de la Santeet de la Recherche Medicale (INSERM), FRANCE

## Abstract

Phylogenic comparisons of the mononuclear phagocyte system (MPS) of humans and mice demonstrate phenotypic divergence of dendritic cell (DC) subsets that play similar roles in innate and adaptive immunity. Although differing in phenotype, DC can be classified into four groups according to ontogeny and function: conventional DC (cDC1 and cDC2), plasmacytoid DC (pDC), and monocyte derived DC (MoDC). DC of Artiodactyla (pigs and ruminants) can also be sub-classified using this system, allowing direct functional and phenotypic comparison of MoDC and other DC subsets trafficking in blood (bDC). Because of the high volume of blood collections required to study DC, cattle offer the best opportunity to further our understanding of bDC and MoDC function in an outbred large animal species. As reported here, phenotyping DC using a monoclonal antibody (mAb) to CD209 revealed CD209 is expressed on the major myeloid population of DC present in blood and MoDC, providing a phenotypic link between these two subsets. Additionally, the present study demonstrates that CD209 is also expressed on monocyte derived macrophages (MoΦ). Functional analysis revealed each of these populations can take up and process antigens (Ags), present them to CD4 and CD8 T cells, and elicit a T-cell recall response. Thus, bDC, MoDC, and MoΦ pulsed with pathogens or candidate vaccine antigens can be used to study factors that modulate DC-driven T-cell priming and differentiation ex vivo.

## Introduction

Recent studies on the phylogeny of the mononuclear phagocyte system (MPS) in humans and mice revealed that phenotypic differences have evolved in subsets of DC that play similar roles in innate and adaptive immunity. A unifying nomenclature has been proposed to show how lineages, defined by expression of different arrays of molecules, can be classified according to ontogeny and function [reviewed in [1]. The cumulative findings indicate that DC can be classified into four subsets: conventional DC (cDC1 and cDC2), plasmacytoid DC (pDC), and monocyte derived DC (MoDC) [1]. Summerfield has proposed to use the same classifications for veterinary species, pointing out where additional phenotypic and ontogenetic information is needed to fully support the classification [2].

Data obtained in Artiodactyla (pigs, cattle, and sheep) support the use of this DC classification system and demonstrate the potential use of these species to further our understanding of DC orchestration of the immune response to infectious agents and vaccines, especially bDC and MoDC. Ex vivo studies in pigs have shown bDC and MoDC can be used to study primary and recall responses to an experimental antigen (Ag) (ovalbumin) and a vaccine (detoxified pertussis toxoid) [3]. This was accomplished by culturing preparations of CD4 and CD8 T cells with bDC and MoDC pulsed with defined Ags. The availability of large quantities of blood, an advantage of using a large animal model, made it possible to obtain enough bDC and MoDC to conduct these studies. Similar use in cattle facilitated comparison of the CD4 T cell response to bovine respiratory syncytial virus using MoDC pulsed with killed and live virus [4]. Since these studies, additional information has been obtained on the phenotype of DC, and on the use of flow cytometry (FC) to characterize CD4 and CD8 T cells responding to Ags presented by DC ex vivo in cattle.

Studies with a mAb we recently developed against CD209, a C-type lectin receptor, show that it is uniquely expressed on myeloid bDC [5], obviating the need to use high speed cell sorting [6, 7] or a panel of mAbs to negatively select bDC for analysis [8]. These studies have also shown CD209 is up-regulated on MoDC and MoΦ (this report) revealing a phenotypic link between these cell subsets.

In this study, we further characterized the phenotype of bDC, MoDc, and MoΦ, and compared their functional capacity to take up, process, and present Ags to CD4 and CD8 T cells. We demonstrate that Ag presentation by CD209^+^ bDC, MoDC, and MoΦ elicits a T-cell recall response to a live *Mycobacterium a*. *paratuberculosis relA* mutant (*Map/relA*) vaccine candidate and a purified *Map* major membrane protein encoded by *MAP* 2121c [9].

## Materials and Methods

### Blood collection and PBMC isolation

Thirteen Holstein steers born and raised in the Washington State University (WSU) dairy herd (n = 6, 4 months of age) or obtained from dairies in Sunnyside central Washington (n = 3, 4 months of age and n = 3, 20 months of age) were the source of blood for different parts of the studies. These animals were obtained for use in other ongoing studies [10]. A 3 year old Holstein steer obtained from the WSU dairy, vaccinated at birth with a *Map/relA* deletion mutant, was used in the Ag recall experiments [11]. Staff were authorized to collect blood from all the animals used in this study. Blood was collected by venipuncture of the jugular vein into acid citrate dextrose. PBMC were isolated as previously described [12, 13] and used for phenotypic analysis of bCD209^+^ DC [5, 14] by flow cytometry (FC) or for generation of MoDC and MoΦ as described below, and also as a source of cells to study recall responses of CD4 and CD8 T cells presented by bDC, MoDC, and MoΦ pulsed with live *Map/relA* or a major membrane protein molecule (MMP) expressed by *Map* [9]. All protocols and procedures were approved by the Washington State University Institutional Animal Care and Use Committee.

### Generation of MoDC and MoΦ

Bovine monocytes were isolated from PBMC using magnetic microbeads coated with an anti-human CD14 antibody (Ab), that reconizes a highly conserved epitope expressed on bovine CD14, per the manufacturer’s instructions (Miltenyi Biotec Ltd., CA) [5]. The average purity of isolated CD14^+^ cells was greater than 98% as determined by FC analysis using an anti-bovine CD14 monoclonal antibody (mAb), CAM36A, (Table 1) [5, 15]. The isolated CD14^+^ monocytes were re-suspended at 6.6 x 10^5^ cells per ml in RPMI 1640 medium with GlutaMAX^TM^ (Life Technologies, CA) supplemented with 10% calf bovine serum (CBS), 1 mM β-mercaptoethanol, 100 units/ml of penicillin G, and 100 μg/ml of streptomycin sulfate.

**Table 1:** Monoclonal antibodies used in present study.

mAb	Isotype	Specificity
H58A	IgG2a	MHC I
PT85A	IgG2a	MHC I
TH14A	IgG2a	MHC II BoLA DR
TH81A5	IgG2a	MHC II BoLA DQ
CAT82A	IgG1	MHC II
ILA11A	IgG2a	CD4
7C2B	IgG2a	CD8
TH97A	IgG2a	CD1b
KD1 AbdSerotec	IgG2a	CD16
HUH73A	IgG1	CD11a
MM12A	IgG1	CD11b
BAQ153A	IgM	CD11c
CAM36A	IgG1	CD14
CAM66A	IgM	CD14
GB25A	IgG1	CD21
CACT114A	IgG2b	CD26
ILA158A	IgG1	CD40
ILA159	IgG1	CD80
ILA190A	IgG1	CD86
LND68A	IgG1	CD163
DH59B	IgG1	CD172a
ILA114A	IgG1	CD205
15.2 Biolegend	IgG1	CD206
209MD26A	IgG2a	CD209

A MoDC growth cocktail containing bovine GM-CSF and IL-4 (Kingfisher Biotech, MN) was used to generate MoDC according to the manufacturer’s instructions. Three milliliters of the cell suspension/MoDC growth cocktail were distributed into each well of a six-well culture plate and cultured at 37°C and 5% CO_2_. On day three, half of the medium was replaced with fresh medium/MoDC growth cocktail without disruption of attached cells. The cells were cultured for an additional three days, and then used in assays as described below.

Two methods were used to generate MoΦ. In one method, PBMC were placed in 10 or 150 mm Petri dishes and incubated for two hours to allow monocyte adherence. Following washing to remove the majority of non-adherent cells, the adherent cells were cultured alone for six days without further treatment. In the second method the adherent cells were cultured in the presence of GM-CSF (100 ng/ml) for six days.

### Morphological analysis

The morphology of MoDC was compared with MoΦ using an EVOS^®^ FL Cell Imaging Microscope (Thermo Fisher Scientific).

### Phenotypic characterization

The expression of CD and MHC II molecules on the surface of monocytes, bDC, MoDC and MoΦ was compared using the mAbs listed in Table 1. Except where indicated, the mAbs were obtained from the Washington State University Monoclonal Antibody Center. http://vmp.vetmed.wsu.edu/resources/monoclonal-antibody-center. Labeling and analysis of monocytes and bDC were performed with PBMC as previously described [5]. In brief, primary mAbs were used at 0.7 μg/10^6^ cells in 200 μL of PBS containing 0.5% horse serum. A polyclonal fluorescein conjugated goat anti-mouse IgG/IgM second step antibody was used to obtain data on the pattern of expression of individual molecules. Isotype specific goat anti-Ig antibodies were used to obtain data on cell subsets: Alexa 647 anti-IgG1, PE-Cy 5.5 anti-IgG2a, PE ant-IgG2b, PE anti-IgM. Following incubation for 15 minutes on ice, the cells were washed with 3 cycles of centrifugation in PBS then re-suspended and incubated with isotype specific goat antibodies conjugated with different fluorochromes second step reagents for an additional 15 minutes washed and re-suspended in 2% formaldehyde. For MoDC labeling, loosely adherent cells were collected following gentle flushing of the culture plates. The remainder of the cells were then detached by incubating the cells in 1 or 10 mL of PBS containing 10 mM EDTA and 10% CBS. The loosely adherent and adherent cells were combined and washed with RPMI 1640 medium by cycles of centrifugation, then labeled with the mAbs as described [5, 11]. For MoΦ, the cells were detached by incubating the cells in PBS/ EDTA/CBS, washed, and labeled with the mAbs.

### Antigens used to study the ex vivo recall response elicited with antigen pulsed bDC, MoDC and MoMΦ.

A candidate *Mycobacterium a*. *paratuberculosis* (*Map*) *relA* deletion mutant (*Map/relA*) vaccine and a candidate *Map* major membrane protein (MMP) were used to study the capacity of bDC, MoDC, and MoMoΦ to process and present antigens (Ag) and elicit a recall response in CD4 and CD8 T cells from a steer vaccinated with *Map/relA* as described below. *Map* is the causative agent of paratuberculosis in cattle (Johne’s disease). Mounting evidence shows it is also the causative agent of Crohn’s disease in humans [16, 17]. The *Map/relA* mutant is one of three deletion mutants developed to test as a candidate vaccine for bovine paratuberculosis [11, 13, 18, 19]. Ongoing studies have shown deletion of *relA* abrogates the capacity of *Map* to establish a persistent infection [11]. It is immune eliminated. A preliminary study has shown it has potential for use in immunotherapy and as a vaccine [19]. MMP is a 35 kD outer membrane protein with a potential for developing a subunit vaccine [9]. It plays a role in cellular invasion.

### Antigen uptake, processing, and presentation

A set of experiments were conducted to compare the capacity of CD209^+^ bDC, MoDC, and MoΦ to take up, process, and present Ag to CD4 and CD8 T cells. As mentioned above, the experiments were designed to detect a recall response to *Map/relA* [11] and MMP [9]. Cultures of *Map/relA* were prepared and used as previously described [11, 13]. The full length MMP protein used in this study was prepared as described previously [20].

### Ag processing and presentation with PBMC

As a positive control, PBMC were prepared as described and distributed in a 6 well tissue culture plate (2 x 10^6^ cell/well) in 5 mL of medium containing live *Map/relA* (ratio 2:1), or MMP (5 μg/ml). One preparation of cells was prepared with medium alone to serve as a negative control. After six days of culture, the cells were collected and processed for FC.

### Ag processing and presentation by bDC in CD14 depleted PBMC

PBMC were depleted of monocytes with magnetic beads coated with an anti-CD14 mAb as described above. FC was used to phenotype and verify the extent of CD14 cell depletion and the presence and frequency of CD209^+^ bDC. CD14 depleted, CD209^+^ PBMC (mdPBMC) were distributed in a 6-well tissue culture plate (10 x 10^6^ cells/well) in 5 ml of medium containing *Map/relA* (ratio 2:1) or MMP (5 μg/ml). One well of cells was prepared with medium alone to serve as a negative control. After six days of culture, the cells were collected and processed for FC.

An identical set of cultures was prepared containing mAbs to MHC I and MHC II (0.5 μg/ml) (Table 1) to determine whether Ag presentation could be blocked. An identical set of cultures was prepared with mAbs to CD209 and CD163 to determine whether Ag presentation involved either receptor, as detected by blocking of Ag presentation.

### Ag processing and presentation by MoDC and MoΦ

Two steps were involved in determining the capacity of MoDC and MoΦ to present Ag to CD4 and CD8 T lymphocytes. Following cell isolation and six day incubation with the DC growth cocktail, the medium was removed and replaced with 5 ml of medium without antigen (negative control) or medium containing MMP (5 μg/ml) or live *Map/relA* (MOI of 10). After three hours of incubation, the medium was removed and the cultures washed twice with warm RPMI to remove free Ag and *Map/relA*. The MoDC and MoΦ were then overlaid with 5 ml of medium containing 10 x 10^6^ freshly mdPBMC from the *Map/relA* vaccinated steer. After six days of incubation, the non-adherent cells were collected and processed for FC.

For negative controls, non-antigen-pulsed MoDC and MoMΦ were overlaid with mdPBMC as above and cultured for 6 days, then processed for FC.

### Flow cytometry

**Fig 1:**
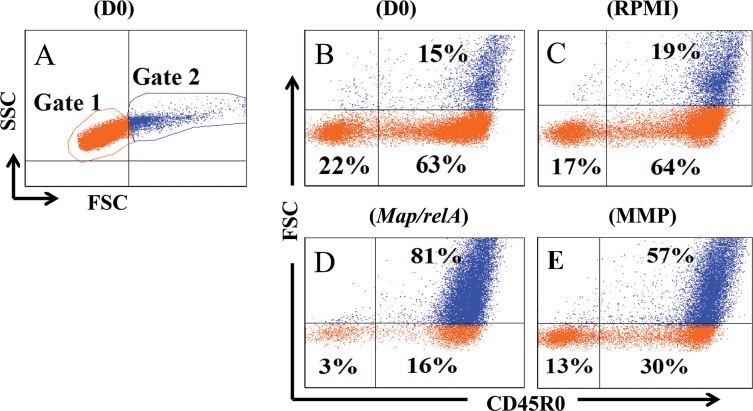
Gating strategy for analysis of data. **A.** Display in SSC vs FSC showing color coding of resting lymphocytes orange (G1) and (G2) monocytes/bDC before culture or activated CD4 and CD8 T cells after culture (illustrated with gated CD4 cells). **B.** Display in FSC vs CD45R0 of CD4 cells at the beginning of culture showing naïve cells lower left quadrant, resting unstimulated memory cells lower right quadrant, and activated proliferating memory T cells upper right quadrant. **C.** Same display of CD4 T cells following culture for 6 days in RPMI only, **D.** Same display showing CD4 T cells cultured with *Map/relA* or **E.** cultured with MMP.

The gating strategy used to analyze the data is shown in Fig 1. The gating strategy was designed to use color coding to distinguish resting non proliferating cells from cells proliferating in response to Ag stimulation and use combinations of mAbs to distinguish cell subsets present in the culture. Cells were first visualized in side light scatter (SSC) vs forward light scatter (FSC). Two electronic gates were then placed on the region containing the majority of small, resting lymphocytes (G1, orange) and the region (G2, blue), containing monocytes, bDC, and enlarged activated lymphocytes in whole PBMC before culture, and activated proliferating T cells after 6 days of culture (Fig 1 A). G2 was selected when collecting data on monocytes and bDC in whole preparations of PBMC. Both gates were used to obtain data on expression of CD molecules expressed on resting and activated proliferating PBMC as previously described [21]. Additional gates were placed on CD4 and CD8 cells to obtain data on the proliferative response of memory T cells following Ag stimulation. As illustrated with a selective gate placed on CD4^+^ T cells and visualized in FSC vs CD45R0, naïve cells, could be distinguished from resting (orange) and activated (blue) memory cells. The relative changes in the proportions of naïve, resting and activated memory CD4 and CD8 T cells was determined with the FCS Express software (Fig 1B–1E). Twenty five thousand events were collected for data analysis with selective gates placed on CD4 and CD8 T cells. FC data were converted to bar graphs for visual comparison of the changes in the relative proportions of naïve, resting and activated memory cells present at the initiation of culture and following culture for 6 days with and without Ag stimulation, rather than presentation in table format.

All FC data were collected with a Becton Dickinson FACS Calibur flow cytometer (BD Immunocytometry Systems, San Jose, CA) and analyzed with FCS Express software (DeNovo Software, Glendale, CA) [5].

## Results

### Generation of MoDC with IL-4/GM-CSF and MoΦ with and without GM-CSF

Monocytes cultured with IL-4 and GM-CSF consistently differentiated into MoDC with a variety of morphological features ranging from well-developed dendrites, when adherent, or fine dendrites when partially or completely detached. Monocytes cultured with or without GM-CSF proliferated and developed and differentiated into MoΦ with morphological features that overlapped those seen in cultures of MoDC consistent with observations by others. Some adherent cells in the population displayed a flattened appearance often seen in some of our cultures of macrophages (data not shown).

### Phenotypic comparison of monocytes, bDC, MoDC and MoΦ

Comparison of expression of phenotypic markers on CD14^+^, CD209^+^ bDC, MoDC, and MoMΦ is summarized in Table 2. Three mAb combinations were used to compare co-expression of CD14, CD21, and CD205 on CD209^+^ bDC. Previous studies have shown CD25 and other activation molecules are only expressed on proliferating blast cells present in G2 as illustrated in FSC vs CD45R0 (Fig 1D and 1E) [5]. Single mAbs were used to determine which molecules were expressed on MoDC and MoMΦ. There were differences in the level of expression of some of the molecules co-expressed by monocytes, bDC, MoDC and MoΦ, but the results were similar to those reported by others [7, 22]. The differences of interest were in expression of CD1b, CD14, CD16, CD21, CD163, CD205, and CD206, CD209 (Table 2). CD1b was expressed on MoDC and MoMΦ but not on monocytes or bDC. CD14 was expressed on monocytes, MoDC, MoΦ, and a subset of CD209^+^ bDC, as described in more detail below. CD16 was expressed on monocytes, MoDC, and MoΦ. Because the Ig isotype of CD16 and CD209 were the same, it was not possible to determine if CD16 is expressed on bDC. As discussed below, CD21 and CD205 were only expressed on subsets of CD209^+^ bDC. CD163 was expressed on a subset of CD209^+^ bDC, monocytes, MoDC, and MoΦ consistant with our recently reported findings [23](data not shown). As noted in our initial studies, CD206 was not expressed on monocytes or CD209^+^ bDC, but was upregulated on MoDC and MoΦ [5].

**Table 2:** Phenotype of monocytes, bDC, MoDC, MoΦ.

Specificity	M	bDC	MoDC	MoΦ
MHC II	+	+	+	+
CD4	-	-	-	-
CD8	-	-	-	-
CD1b	-	-	+	+
CD11a	+	+	+	+
CD11b	+	+	+	+
CD11c	+	+	+	+
CD14	+	Subset	+	+
CD16	+	?	+	+
CD21	-	Subset	-	-
CD26	-	-	-	-
CD40	+	+	+	+
CD80	+	+	+	+
CD86	+	+	+	+
CD163	+	Subset	+	+
CD172a	+	+	+	+
CD205	-	Subset	+	+
CD206	-	-	+	+
CD209	-	+	+	+

- = Negative, + = positive,? = not determined

**Fig 2:**
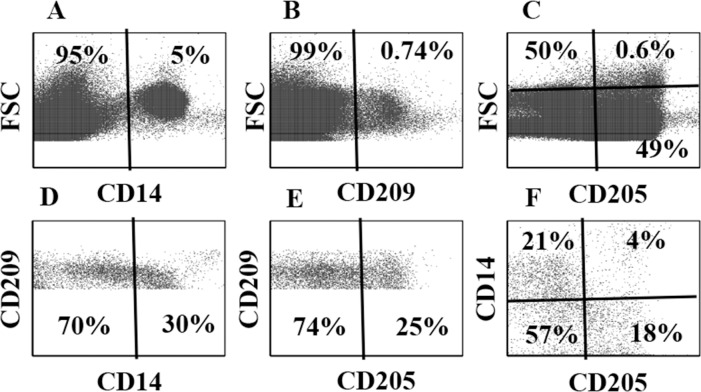
Three mAb comparison of co-expression of CD14 and CD205 on CD209^+^ bDC. **A**, **B**, **C.** Display in FSC vs fluorescence showing proportion of CD14^+^, CD209^+^, CD205^+^ cells present in PBMC with selective gates on **G1** and **G2**. **D.** Display with a selective gate on CD209^+^ bDC showing the proportion of CD209^+^ bDC co-expressing CD14. **E.** The same display with the selective gate on CD209^+^ cells showing the proportion co-expressing CD205. **F.** the same selective gate on CD209^+^ with a display comparing the proportions of CD209^+^ cells expressing only CD14 or CD205 or co-expressing CD14 and CD205.

**Fig 3:**
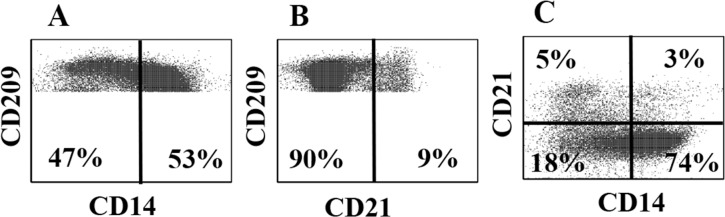
Three mAb comparison of co-expression of CD14 and CD21 on CD209^+^ bDC. **A.** Display showing the proportion of CD14^+^/CD209^+^ bDC with a selective gate on CD209^+^ bDC. **B.** The same display with the selective gate on CD209^+^ cells showing the proportion of CD209^+^ cells co-expressing CD21. **C.** the same selective gate on CD209^+^ with a display comparing the proportions if CD21^+^ cells expressing only CD14 or CD21 or co-expressing CD14 and CD21.

Further analysis of CD209^+^ bDC revealed the population is comprised of subsets with differential expression of CD14, CD21 and CD205. As shown in **Fig 2**, subsets of CD14^+^ and CD205^+^ CD209^+^ bDC were detected with a selective gate on CD209^+^ bDC (Fig 2**D** and 2**E**). With the same gate, comparing CD14 against CD205 revealed 4 different subsets: CD209^+^ alone, CD209^+^/CD14^+^, CD209^+^/CD205^+^ and CD209^+^/CD14^+^/CD205^+^ (Fig 2**F**). As shown in Fig **3**, similar results were obtained comparing expression of CD14 and CD21 on CD209^+^ bDC: CD209 alone, CD209^+^/CD14^+^, CD209^+^/CD21^+^, and CD209^+^/CD14^+^/CD21^+^ (Fig 3**A**, 3**B** and 3**C**).

### Antigen uptake, processing, and presentation by bDC, MoDC, and MoΦ

Previous functional analyses of bovine MoDC used fluorescein-tagged dextran, ovalbumin, and viruses to demonstrate the capacity of MoDC to take up antigens [22, 24] and also used in the mixed lymphocyte reaction to demonstrate a proliferative response [7]. We were interested in extending these observations using a response to candidate vaccines for paratuberculosis (Johne’s disease, JD). Ongoing studies had shown a *relA* deletion mutant of *M*. *a paratuberculosis*, the causative agent of Johne’s disease in cattle elicits and immune response that abrogates the capacity of *Map* to establish a persistent infection, suggesting deletion of relA disrupts the mechanisms used by *Map* to dysregulate the immune response [11]. Unpublished preliminary studies indicated part of the immune response was directed towards MMP, a surface protein under investigation for potential use in a peptide based vaccine. Ability to study the immune response to these candidate vaccines ex vivo would help evaluate their potential efficacy. With this in mind, we compared the capacity of bDC, MoDC, and MoΦ to take up, process and present Ags to T cells and elicit a memory CD4 and CD8 T-cell recall response. A steer maintained from previous studies that was vaccinated with *Map/relA* was used as a source of cells. Ex vivo studies had shown a strong proliferative recall response was elicited following incubation of PBMC with *Map* and *Map/relA* [11]. *Map/relA* was used here to verify that a strong recall response could still be elicited in both CD4 and CD8 T cells and to determine if part of the response was directed towards MMP. MdPBMC were used in these experiments. The premise was that the bDC, with demonstrated functional activity, would not be involved in Ag presentation, since excess Ag would be removed before culturing the mdPBMC with Ag-pulsed MoDC and MoΦ. Bar graphs were used to summarize the FC data and compare the changes in the relative proportion of naïve, resting memory and activated proliferating memory CD4 and CD8 T cells following incubations with Ag pulsed bDC in mdPBMC, MoDC, and MoMΦ. The experiments were repeated six times.

**Fig 4:**
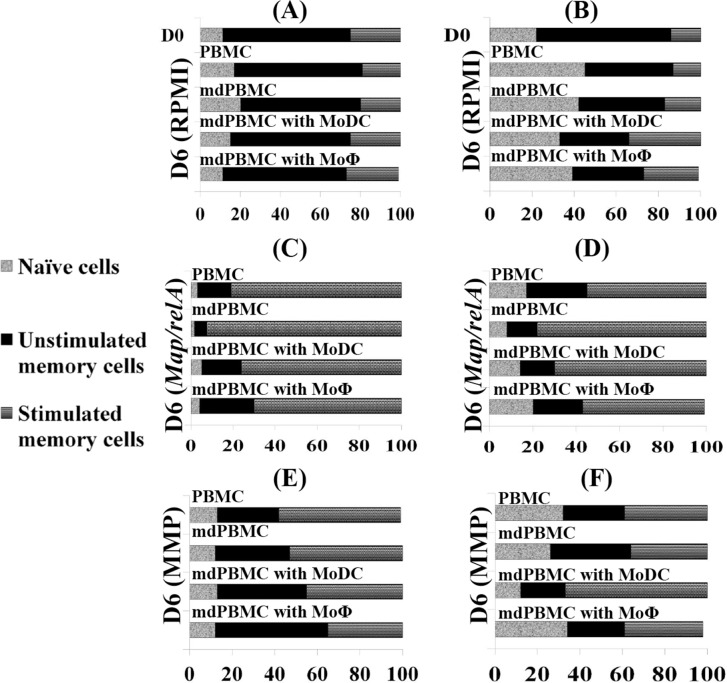
Comparison of the CD4 and CD8 recall responses to *Map/relA* and MMP. **A,** Bar graphs of FC data comparing CD4 T cell responses in whole PBMC at T0 and following culture for 6 days in RPMI alone and mdPBMC cultured in RPMI alone or in the presence of MoDC and MoMΦ not exposed to Ags. **C.** Bar graphs comparing CD4 T cell responses in whole PBMC and in mdPBMC stimulated directly with *Map/relA* for 6 days and in mdPBMC cultured for 6 days with MoDC and MoMΦ pulsed with *Map/relA*. **E.** The same culture conditions with CD4 T cells cultured with MMP directly or with MoDC and MoMΦ pulsed with MMP. **B**, **D**, **F**. Bar graphs of FC data comparing CD8 T cells cultured under the same conditions as CD4 T cells. As noted, naïve, resting memory, and activated memory CD4 and CD8 T cells are distinguished by different shades of grey. Comparisons show there was a clear increase in the proportion of activated memory CD4 and CD8 T cells in cultures stimulated directly with *Map/rel/A* and MMP or through MoDC and MoMΦ pulsed with the Ags. See Fig 1 for gating strategy used to distinguish naïve, resting and activated memory T cells.

In the control cultures, CD4 and CD8 T cells present in PBMC and in mdPBMC did not proliferate without stimulation with *Map/relA* or MMP. **Fig 4**, compare **CD4:** **A 1** and **CD8:** **B 1** PBMC cultured for 6D in RPMI alone: **CD4**: **A 2** and **CD8**: **B 2** mdPBMC cultured for 6D in RPMI alone. Likewise, when mdPBMC were incubated with MoDC and MoΦ alone in the absence of Ag, they did not proliferate. **Fig 4**, compare **CD4:** **A 3** (mdPBMC cultured with MoDC) and **A 4** (mdPBMC cultured with MoΦ) and **CD8**: **B 3** (mdPBMC cultured with MoDC) and **B 4** (mdPBMC cultured with MoΦ). The relative proportions of resting naïve, resting memory, and activated memory cells did not change when cultured under these conditions.

In the experimental cultures, a strong CD4 and CD8 T cell response was elicited with mdPBMC pulsed with *Map/relA* and MMP. **Fig 4**, **CD4:** **C** compare PBMC and mdPBMC cultured directly with *Map/relA* for 6D with mdPBMC cultured with MoDC pulsed with *Map/relA* and mdPBMC cultured with MoΦ pulsed with *Map/relA*. **CD8**: **D** compare PBMC and mdPBMC cultured directly with *Map/relA* for 6D with mdPBMC cultured with MoDC pulsed with *Map/relA* and mdPBMC cultured with MoΦ pulsed with *Map/relA*. **CD4** **E** compare PBMC and mdPBMC cultured directly with MMP for 6D with mdPBMC cultured MoDC pulsed with MMP and mdPBMC cultured with MoΦ pulsed with MMP. **CD8 F** compare PBMC and mdPBMC cultured directly with MMP for 6D with mdPBMC cultured with MoDC pulsed with MMP and mdPBMC cultured with MoΦ pulsed with MMP. The proportion of memory CD4 and CD8 T cells were clearly increased following incubation with MoDC and MoMΦ pulsed with *Map/relA* and MMP. The experiments were repeated six times.

**Fig 5:**
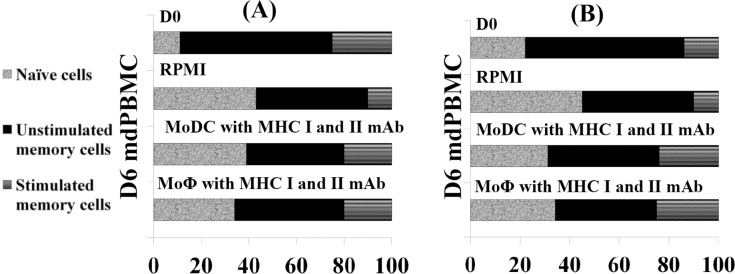
Demonstration showing response to *Map/relA* and MMP is MHC restricted. **A.** CD4 T cells in mdPBMC at T0 and after culture in RPMI for 6 days alone or cultured MoDC or MoMΦ pulsed with *Map/relA* or MMP in medium containing mAbs to MHC I and MHC II. **B**. CD8 T cells in mdPBMC at T0 and after culture in RPMI for 6 days alone or cultured with MoDC or MoMΦ pulsed with *Map/relA* or MMP in medium containing mAbs to MHC I and MHC II.

To determine if the response was MHC restricted, mdPBMC were incubated with *Map/relA* or MMP in the presence of mAbs specific for MHC I and MHC II. As shown in Fig 5, in the presence of mAbs to MHC I and MHC II, Ag presentation to CD4 and CD8 cells by Ag-pulsed MoDC and MoΦ was abrogated. The experiment was repeated three times with similar results.

Following demonstration that the proliferative response was MHC restricted, an additional set of experiments were conducted to determine if CD209 or CD163 were involved in uptake and processing Ags for presentation, as detected by blocking the proliferative response. The response to Ag presented by MoDC and MoΦ pulsed with *Map/relA* and MMP was unaffected by inclusion of mAbs to CD209 and CD163 in the culture medium (data not shown).

## Discussion

DC phenotyping, using mAbs to differentially expressed non-lineage molecules in cattle and pigs has provided evidence that the ontogeny of DC subsets in these species is similar to DC ontogeny in humans and mice and demonstrates the value of these large, outbred species in studying the role of DC in orchestration of the immune response [2]. Due to the large blood volume of Artiodactyls, abundant monocytes can be isolated to generate MoDC, facilitating investigation of DC antigen uptake and processing through different receptors, signaling pathways that drive T cell differentiation, cytokines secreted during Ag presentation, primary and recall T-cell responses to whole pathogens and candidate vaccines, and importantly, the functional activity of CD4 and CD8 T cells proliferating in response to Ags presented by MoDC [3, 4]. To characterize bovine DC in this model, we developed a mAb to CD209 to determine if we could identify the major population of myeloid DC in blood more directly and compare the phenotype of bDC phenotype with that of MoDC and MoΦ [5]. We were also interested in extending and comparing information on the functional activity of these populations in Ag processing and presentation of Ag peptides to CD4 and CD8 T cells using a live attenuated candidate vaccine, *Map/relA* and a candidate major cell surface expressed membrane protein (MMP).

CD209 (DCSIGN, ICAM-3-grabbing non-integrin) is a multifunctional receptor. It plays a role in DC tissue trafficking [25] as well as development of innate and adaptive immunity [26]. It appears on monocytes recruited to lymph nodes by LPS and microbial antigens, and potentiates their capacity to take up and present antigens to naïve T cells [27, 28]. CD209 initially garnered interest due to findings that it is used by the human immunodeficiency virus and *M*. *tuberculosis* for uptake, and is important in the pathogenesis of these diseases [29, 30]. Subsequent studies in mice and humans showed it is highly expressed on bDC, MoDc and some tissue MoΦ [31, 32]. Although CD209 is not included in the set of molecules currently used to define the subsets of DC in humans or mice [1], we sought to examine CD209 expression on DC in cattle. Our initial studies described the development of the mAb and characterization of the phenotype of circulating bDC in PBMC and MoDC [5]. In the present study, we compared the phenotype of bDC with monocytes, MoDC and MoΦ in an effort to reveal possible functional distinctions between these subsets. Repeated screening of PBMC revealed CD209 is expressed on all myeloid DC present in blood. Comparison of expression of CD209 with other CD molecules revealed it is comprised of multiple subsets as detected by comparison of expression of CD14, CD21, and CD205 on CD209^+^ bDC. Comparison of expression of CD14 and CD205 on CD209^+^ bDC revealed the presence of 4 populations: a large CD209^+^/CD14^-^/CD205^-^ population and smaller subsets of CD209^+^/CD14^+^/CD205^-^, CD209^+^/CD14^-^/CD205^+^, and CD209^+^/CD14^+^/CD205^+^ cells. Comparison of CD14 and CD21 on CD209^+^ bDC revealed a similar complexity: a large CD209^+^/CD14^-^/CD21^-^ population and smaller subsets of CD209^+^/CD14^-^/CD21^+^, CD209^+^/CD14^+^/CD21^-^, CD209^+^/CD14^+^/CD21^+^ cells. Five animals were used to obtain the results with CD21. Six animals were used to obtain the results with CD205. Further studies are needed to determine if there are any functional differences between the mAb defined subsets. Of interest, comparing the proportion of CD209^+^/CD14^+^ CD209^+^ cells in bDC examined in this study suggests the relative proportion may vary. Whether this reflects the presence of DC in the process of differentiating into DC with the characteristics of MoDC needs to be examined in further detail. The main message from this part of the study is that it appears the indirect methods used previously to isolate bDC for comparative studies most likely were missing some of the CD209^+^ subsets [6, 7]. It is clear that further studies are needed to clarify whether one or both CD209^+^/CD14^+^ and CD209^-^/CD14^+^ subsets proliferate in response to stimulation with GM-CSF and IL-4. The present study shows depletion with CD14 leaves only CD209^+^ bDC subsets in blood and that they have the capacity to process and present Ags to CD4 and CD8.

Further comparisons of bDC with MoDC and MoΦ revealed all 3 populations express CD209. A subset of CD209^+^ bDC express CD163 and [Just published in a follow up study [23]]. MoDC and MoΦ express CD1b, CD163, and CD206. As mentioned, CD21 and CD205 are only expressed on subsets of CD209^+^ bDC (Table 2).

A final observation of interest in phenotyping is that, in contrast to MoDC generated from monocytes in humans [33], expression of CD14 is not down regulated on MoDC. Expression persists on MoDC and also on MoΦ. Repeated experiments document this difference in expression of CD14 on human and bovine MoDC.

While numerous studies on Ag processing and presentation by bovine MoDC have been conducted, few studies have been conducted to determine the capacity of bovine bDC to take up and present Ag. One study showed the cytokine gene profile of MoDC pulsed with a live bovine respiratory disease virus differed from that of MoDC pulsed with an inactivated virus preparation, suggesting signaling in the recall response could lead to a different effector function outcomes [4]. Unfortunately, the lack of a method to examine the effector activity of CD4 T cells proliferating in response to viral Ag presented by MoDC left the question unanswered. A more recent report, where high speed cell sorting was used to negatively select a CD11c^+^/CD205^+^ population of bDC, described two populations with similar capacity to take up antigen but with different capacities to activate T cells [7]. Similar to the earlier study, no method was available to study effector activity of cells responding to bDC pulsed with Ag.

As mentioned above, in addition to gaining information on the phenotype of bDC, MoDC, and MoΦ, we were interested gaining further information on their functional activity using an ex vivo model to examine the response of CD4 and CD8 T cells responding to Ags processed and presented by bDC, MoDC, and MoMΦ. Recent progress in the study of the immune response to *Map* provided an unique opportunity to use a candidate vaccine *Map/relA* mutant and a major membrane protein (MMP) to obtain initial data on an ex vivo model. Previous studies showed deletion of *relA* abrogates the capacity of the mutant to establish a persistent infection, allowing immune clearance [11]. Earlier studies comparing the proliferative response to MMP or whole organisms in PBMC from steers infected with *Map* or *Map/relA* showed MMP elicited a recall response similar to stimulation with *Map* and *Map/relA* (unpublished observation). The first questions we wanted to answer were: 1) Can Ag-pulsed bDC, present in PBMC, elicit a recall response equivalent or greater than that observed with PBMC containing monocytes? 2) If so, is the response MHC restricted and/or restricted through CD209 or CD163? 3). Are there any differences in the capacities of bDC, MoDC, and MoΦ, pulsed with Ag, to elicit a recall response? 4) Are there any differences in the proliferative responses elicited by bDC, MoDC, and MoΦ, pulsed with *Map/relA* or MMP?

Although the ideal comparative experiment would have been with bDC isolated from PBMC, we chose to start by comparing the proliferative response of PBMC with that of mdPBMC, since the mdPBMC containing bDC provided the first opportunity to determine if bDC pulsed with Ag can elicit a T-cell recall response. From a methodological view, demonstration of functional activity without the need for purification, would simplify bDC studies. The proliferative response of PBMC and mdPBMC to *Map/relA* and MMP were equivalent, suggesting bDC were the major APC presenting Ags in previous studies with irradiated PBMC. The proliferative response of mdPBMC was completely blocked in the presence of mAbs to MHC I and MHC II, indicating the response is MHC restricted. Further studies are needed to examine the effect of MHC I and MHC II used alone on isolated sets of CD4 and CD8 T cells. Inclusion of mAbs to CD209 and CD163 had no effect on Ag processing and presentation, suggesting different receptors were involved in Ag uptake or that binding of the mAbs did not abrogate receptor function.

The rationale for using mdPBMC and not PBMC depleted of both monocytes and bDC for the following studies was based on the premise that the bDC present in the mdPBMC would not be involved in Ag presentation, since excess Ags would be removed before incubating the mdPBMC with the Ag-pulsed MoDC and MoΦ. There was no proliferative response when mdPBMC were incubated with MoDC or MoΦ not pulsed with Ags. Comparison of the proliferative response elicited by MoDC and MoΦ pulsed with *Map/relA* and MMP revealed they were equally capable of eliciting a recall response. As with bDC and MoDC, MoΦ pulsed with Ag also elicited comparable recall responses in CD4 and CD8 T Cells.

Of importance, the comparative study of the response to *Map/relA* and MMP answered the question concerning the potential of MMP as a candidate subunit vaccine component for JD. The long term objective of the ongoing studies has been to develop ex vivo platforms to obtain data predictive of the immune response to candidate vaccines in vivo, especially peptide based vaccines. The study showed MMP elicited CD4 and CD8 T cell recall responses equivalent to those elicited by *Map/relA*. Further studies are underway to determine the potential of MMP for developing a subunit vaccine. Unlike wild-type *Map*, *Map/relA* cannot persist, and is subject to immune clearance. The strong response to MMP indicates this is a major component of the immune response to *Map*, and may contribute to the inability of the mutant to establish a persistent infection by blocking the pathways used by *Map* to disrupt development of protective immunity.

In summary, we extended findings on the phenotype and function of bovine bDC, MoDC, and MoΦ, and characterized their potential use in development of an ex vivo large animal model to study the immune response to pathogens and candidate vaccines, using a relevant live attenuated candidate vaccine and a candidate peptide. In cattle, CD209 identifies the major myeloid population of DC in blood and provides a phenotypic lineage link with MoDC and MoΦ. Comparison of their functional activity revealed they are equally capable of processing and presenting Ag to T cells and eliciting CD4 and CD8 T cell recall responses. The latter findings indicate it should be possible to exploit the use of a large animal species to study factors that modulate antigen processing and the capacity of DC to drive differentiation of CD4 and CD8 T cells along different pathways. Using bovine DC, it should also be possible to study the specificity and effector activity of CD4 and CD8 T cells proliferating in response to cytokines secreted by DC at the time of Ag presentation. Sequential re-stimulation can be used to expand Ag specific clones for analysis of functional effector activity.
