# Correction: Phenotype and Function of CD209^+^ Bovine Blood Dendritic Cells, Monocyte-Derived-Dendritic Cells and Monocyte-Derived Macrophages

**DOI:** 10.1371/journal.pone.0171059

**Published:** 2017-01-25

**Authors:** Kun Taek Park, Mahmoud M. Elnaggar, Gaber S. Abdellrazeq, John P. Bannantine, Victoria Mack, Lindsay M. Fry, William C. Davis

The second author’s name appears incorrectly in the published article. The correct name is: Mahmoud M. Elnaggar. The correct citation is: Park KT, Elnaggar MM, Abdellrazeq GS, Bannantine JP, Mack V, Fry LM, et al. (2016) Phenotype and Function of CD209^+^ Bovine Blood Dendritic Cells, Monocyte-Derived-Dendritic Cells and Monocyte-Derived Macrophages. PLoS ONE 11(10): e0165247. doi:10.1371/journal.pone.0165247
